# Upregulated IGFBP3 with Aging Is Involved in Modulating Apoptosis, Oxidative Stress, and Fibrosis: A Target of Age-Related Erectile Dysfunction

**DOI:** 10.1155/2022/6831779

**Published:** 2022-02-03

**Authors:** Daoyuan Hu, Yunlong Ge, Yubin Cui, Ke Li, Jialiang Chen, Chi Zhang, Qiwei Liu, Lizhao He, Weijun Chen, Jun Chen, Cheng Hu, Hengjun Xiao

**Affiliations:** ^1^Department of Urology, The Third Affiliated Hospital, Sun Yat-sen University, Tianhe Road 600#, Guangzhou, 510630, China; ^2^Department of Infertility and Sexual Medicine, The Third Affiliated Hospital, Sun Yat-sen University, Tianhe Road 600#, Guangzhou, 510630, China

## Abstract

Aging has been deemed the primary factor in erectile dysfunction (ED). Herein, age-related changes in the erectile response and histomorphology were detected, and the relationship between aging and ED was investigated based on gene expression levels. Thirty male Sprague–Dawley (SD) rats were randomly divided into 6 groups, and intracavernous pressure (ICP) and mean arterial pressure (MAP) were measured. Subsequently, the corpus cavernosum (CC) was harvested and prepared for histological examinations of apoptosis, oxidative stress (OS), and fibrosis. Then, the microarray dataset (GSE10804) was analyzed to identify differentially expressed genes (DEGs) in ED progression, and hub genes were selected. In addition, aged CC smooth muscle cells (CCSMCs) were isolated to evaluate the function of the hub gene by siRNA interference, qRT–PCR, immunofluorescence staining, enzyme-linked immunosorbent assay, western blot analysis, CCK-8 assay, EdU staining, and flow cytometry approaches. The ICP/MAP and smooth muscle cell (SMC)/collagen ratios declined with aging, while apoptosis and OS levels increased with aging. The enriched functions and pathways of the DEGs were investigated, and 15 hub genes were identified, among which IGFBP3 was significantly upregulated. The IGFBP3 upregulation was verified in the CC of aging rats. Furthermore, aged CCSMCs were transfected with siRNA to knock down IGFBP3 expression. The viability and proliferation of the CCSMCs increased, while apoptosis, OS, and fibrosis decreased. Our findings demonstrate that the erectile response of SD rats declines in parallel with enhanced CC apoptosis, OS, and fibrosis with aging. Upregulation of IGFBP3 plays an important role; furthermore, downregulation of IGFBP3 improves the viability and proliferation of CCSMCs and alleviates apoptosis, OS, and fibrosis. Thus, IGFBP3 is a potential therapeutic target for age-related ED.

## 1. Introduction

Erectile dysfunction (ED), a common sexual dysfunction defined as the inability to achieve or maintain sufficient penile erection satisfactory for sexual intercourse, severely affects the quality of life of millions of individuals [1]. Epidemiological data indicate that more than 100 million people worldwide are affected by ED, and this figure is expected to exceed 300 million by 2025 [2]. Aging is a key risk factor for ED [3]. A recent study showed that the incidences of ED in individuals under 30, 30 to 39, 40 to 49, 50 to 59, 60 to 69, and ≥70 years of age were 20.86%, 25.30%, 40.48%, 60.12%, 79.10%, and 93.72%, respectively [4]. Furthermore, in older males, sexual activity remains important for the maintenance of relationships [5]. Thus, it is necessary to thoroughly investigate the connection between aging and ED.

The specific mechanism of age-related ED has not been elucidated, but ED is generally considered to be the result of multiple factors, such as the phenotypic transformation of corpus cavernosum (CC) smooth muscle cells (CCSMCs), endothelial dysfunction, and decreased androgen levels [6]. The various factors interact to cause degeneration and fibrosis of CCSMCs, resulting in organic lesions in penile tissue that are directly responsible for the occurrence of age-related ED [7]. Recently, the etiology of age-related ED has been explored from different perspectives to support future clinical transformation. Wang et al. [8] found that increases in apoptosis, fibrosis, and oxidative stress (OS) occurred in rats with age-related ED and may be the targets of losartan. Bragina et al. [9] found that the renin-angiotensin system (RAS) is involved in age-related ED and that the activity of the ACE-Ang II-AT1 axis in the CC is enhanced, which is positively correlated with fibrosis and inflammation of the CC. Age-related ED can be ameliorated by alleviating TGF-*β*-induced fibroblast-to-myofibroblast transition via suppressing of the ACE-Ang II-AT1 axis. Kaya-Sezginer and Gur [10] found that endothelial dysfunction caused by low-grade inflammation may play a pathological role in ED, thus providing a meaningful target for the treatment of ED. Macit et al. [11] showed that caloric restriction and physical exercise can restore endothelial and smooth muscle cells (SMCs) in the CC by decreasing apoptosis and improving endothelial function.

At present, phosphodiesterase-5 inhibitors (PDE5Is), which are classic oral drugs, are still the first-line treatments for age-related ED; however, the overall efficacy of PDE5Is in age-related ED is only approximately 65.7% [12]. Moreover, PDE5Is have contraindications; for example, they cannot be used with nitrates, which are necessary for older individuals with cardiocerebrovascular diseases. Such contraindications restrict the application of PDE5Is. Other treatments for ED include intracavernous injection (ICI), vacuum erectile device (VED) use, low-intensity extracorporeal shockwave therapy (LESWT), penile prosthesis implantation (PPI), and other approaches, which have curative effects to certain degrees; however, long-term application can still be limited by side effects or potential complications [13]. Hence, novel and more effective diagnostic and treatment targets are urgently needed.

Herein, an animal model was established in male rats of different ages, and the differences in erectile response and corresponding histomorphological changes were explored. We found that erectile function and the SMC/collagen ratio of the CC decreased with aging, while apoptosis and OS levels increased. To interpret the causes of changes at the genetic level, bioinformatics analysis was used to evaluate a dataset from the Gene Expression Omnibus (GEO) database, and the identified hub gene insulin-like growth factor binding protein-3 (IGFBP3) was found to be significantly upregulated in ED samples. The IGFBP3 upregulation with aging was further verified in rat CC. Then, aged rat CCSMCs were isolated and transfected with siRNA to knock down the expression of IGFBP3. Subsequently, either the reductions in viability and proliferation or the increases in apoptosis, OS, and fibrosis of the CCSMCs were attenuated, indicating the prognostic value and therapeutic potential of IGFBP3 for age-related ED.

## 2. Materials and Methods

### 2.1. Animals

Adult male Sprague–Dawley (SD) rats were purchased from the SYSU Laboratory Animal Center (Guangzhou, China). These rats (*N* = 30) were divided into six groups (5/group) according to age: the 4-month-, 8-month-, 12-month-, 16-month-, 20-month-, and 24-month-old groups. All animals were ear-tagged for identification and housed individually in a room set at 22–24°C with a 12 h light/dark cycle. All rats were provided regular rodent chow and tap water ad libitum. All experimental protocols were approved by the Committee of Animal Care and Use at Sun Yat-sen University.

### 2.2. Evaluation of Erectile Function

After the animals reached the designated ages, erectile function was assessed as we previously reported [14]. Briefly, the rats were anesthetized with 2% pentobarbital sodium (40 mg/kg). For the detection of the mean arterial pressure (MAP), an incision was made along the midline neck to expose the carotid arteries. The carotid artery was cannulated with a tube filled with heparin for recording of the MAP. Then, the cavernous nerves were exposed through a low abdominal incision. After that, the skin of the penis was stripped off, and a 25-gauge needle was implanted into the CC. Then, a delicate stainless steel bipolar electrode that was connected to a multichannel signal producer (BL-420F, Taimeng, China) was used to stimulate the nerves under the following parameters: 5 V, 25 Hz, pulse width of 5 ms, duration of 1 min, and interval period of 2–3 min. The MAP and the intracavernous pressure (ICP) were recorded, and the erection function was determined using the ratios of ICP to MAP. After measurements were obtained, the rats were euthanized and the penile tissues were harvested for subsequent analysis.

### 2.3. Hematoxylin and Eosin (HE) Staining

The collected CC samples were fixed in 4% paraformaldehyde for 24 h at 4°C and then embedded in paraffin blocks. HE staining was conducted. In brief, the sections were deparaffinized with xylene I and xylene II for 20 min each, washed with various concentrations of ethanol for 5 min each, rinsed with tap water, stained with hematoxylin solution for 3–5 min, and differentiated with 0.5–1% hydrochloric acid ethanol for 30 s after washing. After that, the sections were treated with hematoxylin and Scott's tap water bluing reagent and rinsed with tap water. Then, the sections were stained with eosin dye for 5 min, conventionally dehydrated, cleared, and mounted. Afterward, the sections were imaged with a microscope.

### 2.4. Masson Staining

The sections of CC were dewaxed with xylene I and xylene II for 20 min each, washed with various concentrations of ethanol for 5 min each, rinsed with tap water, and dyed with Weigert's iron hematoxylin for 20 min. Then, the sections were washed and dyed with Biebrich scarlet-acid fuchsin. Subsequently, the slices were treated with phosphomolybdic-phosphotungstic acid, aniline blue, 1% glacial acetic acid, dried, and mounted in sequence. The ratios of smooth muscle to collagen were determined using an upright optical microscope and ImageJ software.

### 2.5. Terminal Deoxynucleotidyl Transferase dUTP Nick-End Labeling (TUNEL) Staining

A TUNEL staining kit (Invitrogen, USA) was used to evaluate CC cell apoptosis. CC tissues were deparaffinized with xylene twice (5 min each) after slicing. Then, the sections were dehydrated with gradient ethanol, rinsed with distilled water, and incubated in proteinase K/10 mM Tris solution for 15–30 min at 37°C. TUNEL detection solution was applied to each section, and the sections were incubated for 60 min at 37°C in the dark. After rinsing with phosphate-buffered saline (PBS) three times, the sections were sealed with an antifluorescence quenching mounting reagent. Then, each slice was incubated with 4′,6-diamidino-2-phenylindole (DAPI) at room temperature for 10 min, and photographs were taken under a fluorescence microscope.

### 2.6. OS Level Detection

OS in the CC tissues and CCSMCs was measured according to the levels of malondialdehyde (MDA) and superoxide dismutase (SOD) with test kits (Beyotime Biotechnology, China) according to the manufacturer's instructions. The CC MDA levels and SOD activity were normalized to the total protein concentration of the samples. The reactive oxygen species (ROS) activity of CCSMCs was detected by the fluorescence probe method according to the protocol of the test kit (Beyotime Biotechnology, China).

### 2.7. Bioinformatics Analysis

The microarray dataset GSE10804 consisting of ED and non-ED sample data was downloaded from the Gene Expression Omnibus (GEO) to find candidate genes related to ED. The Limma R package was used to identify differentially expressed genes (DEGs). Genes with a *P* value < 0.05 and a ∣log2(fold change, FC) | >1 were considered significant DEGs. After that, functional enrichment analyses were performed, including Gene Ontology (GO) and Kyoto Encyclopedia of Genes and Genomes (KEGG) analyses, through the Database for Annotation, Visualization and Integrated Discovery (DAVID) online database, and *P* < 0.05 was taken to represent statistical significance. In addition, a protein–protein interaction (PPI) network was established and simplified with the Search Tool for the Retrieval of Interacting Genes/Proteins (STRING) and Cytoscape. This network was used to predict the molecular basis of the disease. The interactions with an average score greater than 0.4 were considered significant. Finally, the hub genes were analyzed and visualized with the cytoHubba plugin of Cytoscape with the maximal clique centrality (MCC) method.

### 2.8. CCSMC Isolation and Culture

The penises from 24-month-old rats (5 per time) were collected and washed several times with ice-cold PBS containing 100 U/mL penicillin and 100 mg/mL streptomycin to remove blood. Then, the tissues except the CC were removed, and the remaining CC tissue was cut into 1 to 2 mm^3^ segments. Afterward, the segments were placed uniformly at the bottom of a sterile 25 cm^2^ culture flask (Corning, USA) containing approximately 2 mL of high-glucose (HG) Dulbecco's modified Eagle's medium (DMEM, Invitrogen) supplemented with 10% fetal bovine serum (FBS, Invitrogen), 100 U/mL penicillin, and 100 mg/mL streptomycin. The tissue samples were shaken and incubated at 37°C with 0.2% collagenase II for 1.5–2 h. Subsequently, the cell suspension was centrifuged (1000×g, 10 min), and the supernatant was discarded. The pellet was resuspended in medium and incubated with 0.25% trypsin for 30 min. Then, the digestion was terminated with serum-containing medium and the suspension was passed through a cell strainer. After that, the cell suspension was gently pipetted and centrifuged (1000×g, 10 min). The pellet was resuspended in a culture flask at 37°C in a humidified atmosphere of 95% air and 5% CO_2_. The medium was replaced with new DMEM containing 10% FBS 2–3 days later. The cells were identified through *α*-actin using immunofluorescence staining and cytomorphology using an inverted microscope. Cells in the third or fourth passage were used for subsequent experiments.

### 2.9. Quantitative Real-Time PCR (qRT–PCR)

The TRIzol reagent kit (Thermo Fisher Scientific, USA) was applied for the extraction of total RNA from rat CC tissues. The RNA was reverse-transcribed using a PrimeScript® RT Reagent Kit (Takara, Japan). After that, qRT–PCR was performed using a StepOne Plus System (Applied Biosystems, USA) with a SYBR Premix ExTaq II (TliRNase HPlus) kit (Takara, Japan). The 2^−*ΔΔ*Ct^ method was applied to process the experimental data. The expression of related genes was normalized to the quantity of GAPDH in each individual sample to correct for sample variability. All primers are listed in Table 1.

### 2.10. Western Blot Analysis

Tissue proteins were extracted by RIPA lysis buffer (Cell Signaling Technology, USA) containing a protease inhibitor cocktail (KeyGEN BioTECH, China). The protein concentration was determined by the BCA protein assay (Beyotime, China). After that, the proteins were isolated by SDS–PAGE and then passed on to a PVDF membrane (Millipore, USA). The membrane was blocked with 5% bovine serum albumin at room temperature for 1 h and then incubated with primary antibodies against collagen I (Abcam, USA), matrix metalloproteinase 9 (MMP9) (Abcam, USA), IGFBP3 (Abcam, USA), B-cell lymphoma-2 (Bcl-2) (Abcam, USA), Bcl-2-associated X protein (Bax) (Abcam, USA), and GAPDH (Abcam, USA). The membrane was washed and soaked with appropriate secondary antibodies at room temperature for 1 h. The protein bands were visualized using enhanced chemiluminescence (ECL) reagents (Millipore, USA) and imaged with an imaging system (Tanon, China). The GAPDH signal was used as a loading control.

### 2.11. Immunohistochemistry and Immunofluorescence

Sections were dewaxed in xylene for 15 min to remove embedding medium, rehydrated in a descending gradient of ethanol solutions (100%, 95%, 90%, 70% 50%, and 30%), and placed in water for 5 min each. Cells were fixed in 4% formaldehyde (freshly prepared from paraformaldehyde) in PBS (pH 7.4) for 30 min and permeabilized for 15 min with 0.5% Triton X-100 in PBS. After blocking with serum for 30 min, the sections and cells were incubated with antibodies including anti-IGFBP3 (Abcam, USA), anti-*α*-actin (Abcam, USA), anti-collagen I (Abcam, USA), and anti-MMP9 (Abcam, USA) at 4°C overnight. The sections and cells were washed with PBS and incubated with appropriate secondary antibodies at room temperature for 50 min. After rinsing with PBS, DAPI was applied to locate the cell nucleus. The stained sections and cells were examined and photographed using a microscope (Nikon, Japan).

### 2.12. siRNA Interference

Rat IGFBP3 siRNA and nonsilencing control siRNA were obtained from Guangzhou RiboBio Co., Ltd. (RiboBio, China). Primary CCSMCs were isolated from 24-month-old SD rats and cultured in DMEM (Invitrogen) supplemented with 10% FBS (Invitrogen) containing 100 U/mL penicillin and 100 mg/mL streptomycin. The cells were maintained at 37°C in a humidified atmosphere containing 5% CO_2_. For IGFBP3 knockdown, CCSMCs were transiently transfected with 50 nM IGFBP3 siRNAs using riboFECT™ CP (Thermo Fisher, USA) according to the manufacturer's instructions.

### 2.13. Cell Viability and Proliferation Assay

A Cell Counting Kit-8 (CCK-8, APExBio, USA) was used to determine cell viability and proliferation. After the specified processing was performed for each group, 1 × 10^4^ cells in 100 *μ*L of medium were plated in 96-well plates. CCK-8 reagent (10 *μ*L) was added, and the cells were then cultured at 37°C for 2 h. In addition, a microplate reader (Thermo VarioskanLUX, USA) was used to measure the absorbance at a wavelength of 450 nm. The percentage of cell viability was subsequently obtained by calculating the mean optical density (OD) in each group. The experiments were performed in triplicate. An EdU assay was performed using an EdU Cell Proliferation Kit with Alexa Fluor 594 in accordance with the manufacturer's instructions (RiboBio, China). CCSMCs were cultured in HG DMEM (Gibco, USA) containing EdU at 37°C for 48 h. Then, the cells were fixed with 4% formaldehyde for 20 min and treated with glycine for 5 min. After washing twice, each well was treated with 200 *μ*L of 1x Apollo reaction cocktail for 20 min. Subsequently, nuclear DNA was stained with DAPI, and the cells were examined with a fluorescence microscope (Nikon, Japan).

### 2.14. Flow Cytometric Apoptosis Assay

The apoptosis levels of CCSMCs were detected using an Annexin V-FITC/PI kit (Nanjing KeyGen Biotech Co., Ltd., China) as previously described [15]. Briefly, CCSMCs were cultured in 6-well plates (2 mL/well) at a concentration of 1 × 10^6^ cells/mL. After washing, the CCSMCs of each group were collected. Next, the cells were mixed with 400 *μ*L of 1x Annexin V containing 5 *μ*L of Annexin V-FITC staining fluid and then incubated in the dark at 4°C for 15 min. After that, 10 *μ*L of PI staining fluid was added, and the cells were incubated for 5 min. Finally, flow cytometry (BD Biosciences, USA) was introduced to analyze the apoptosis levels of CCSMCs. The total apoptosis rate was calculated as the early apoptotic rate plus the later apoptotic rate.

### 2.15. Statistical Analysis

The results are presented as the means ± standard deviations (SD) and were analyzed using SPSS version 20. Statistical analysis was performed using one-way ANOVA and Dunnett's tests. The results were considered to be significant at *P* < 0.05.

## 3. Results

### 3.1. The Erectile Response of SD Rats Decreased with Aging

**Figure 1:**
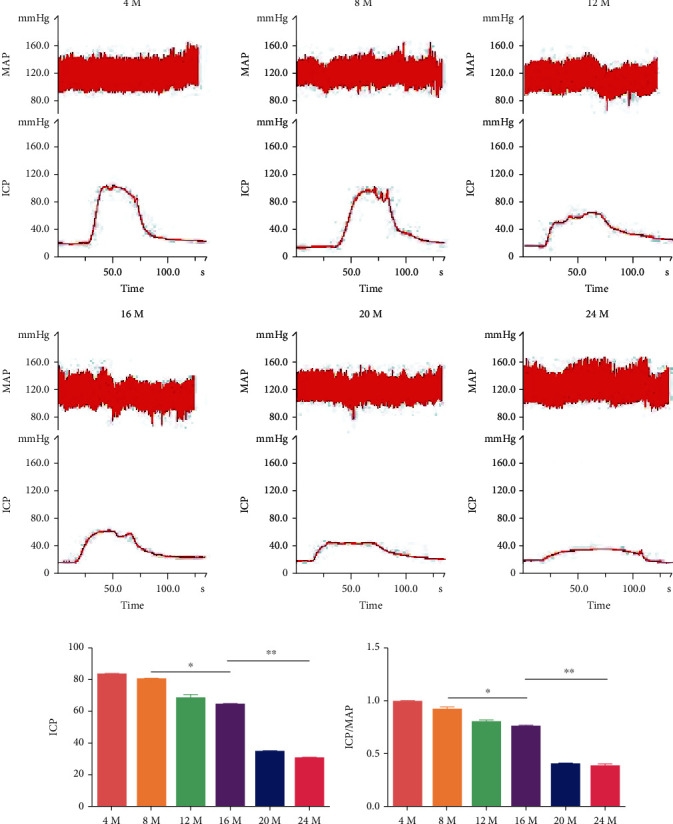
Erectile response as indicated by the ICP/MAP ratio. (a) Representative images of MAP (top) and ICP (bottom) in the 4-month-, 8-month-, 12-month-, 16-month-, 20-month-, and 24-month-old groups. (b, c) Erectile function as indicated by the ICP and ICP/MAP values. Each bar represents the means ± SD, *n* = 5 rats per group. ^∗^*P* < 0.05 and ^∗∗^*P* < 0.01 compared with the 16-month-old group.

ICP analysis showed that the ICP/MAP ratios were 0.83 ± 0.02 in the 4-month-old group, 0.80 ± 0.02 in the 8-month-old group, 0.66 ± 0.02 in the 12-month-old group, 0.64 ± 0.01 in the 16-month-old group, 0.34 ± 0.02 in the 20-month-old group, and 0.32 ± 0.02 in the 24-month-old group (Figure 1(a)). The ICP and ICP/MAP values decreased with aging; however, there were no significant differences between the 4-month- and 8-month-old groups. The values in the 16-month-old group were lower than those in the 8-month-old group, while the ICP/MAP values in the 24-month-old group were the lowest (Figures 1(b) and 1(c), *P* < 0.05).

### 3.2. Apoptosis and OS Levels within the CC Increased with Aging

**Figure 2:**
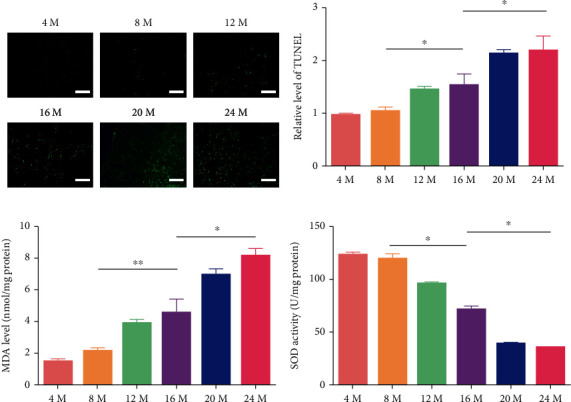
Apoptosis and OS levels of the CC with aging. (a) Fluorescence images of the CC following TUNEL staining (green) (magnification ×200). (b) The percentage of TUNEL-positive CC cells. (c, d) MDA levels and SOD activity normalized to the total protein concentrations of penile samples. The data are represented as the means ± SD for *n* = 3 per group. ^∗^*P* < 0.05 compared with the 16-month-old group.

CC apoptosis and OS levels were detected by TUNEL staining and enzyme-linked immunosorbent assay. The results showed that apoptosis levels increased with aging. The fluorescence intensity in the 16-month-old group was higher than that in the 8-month-old group, while the fluorescence intensity in the 24-month-old group was the highest (Figures 2(a) and 2(b), *P* < 0.05). The activity of SOD decreased with aging, while the activity of MDA decreased, especially in the 8-month-, 16-month-, and 24-month-old groups (Figures 2(c) and 2(d), *P* < 0.05).

### 3.3. Collagen Levels Increased, SMC Content and Vessel Quantities Decreased, and Histomorphology Changed with Aging

**Figure 3:**
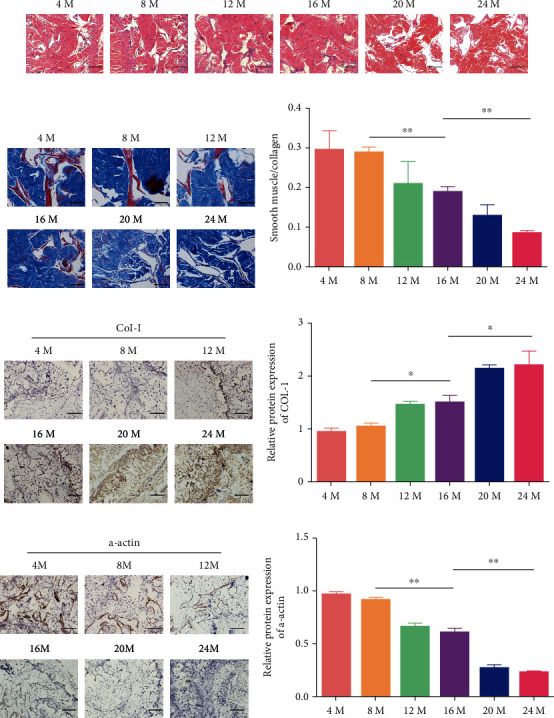
Representative images of histomorphological changes in the CC (magnification 200x). (a) Age-related histomorphological changes in the CC. (b, c) Images and ratios of SMCs (red) and collagen (blue) in each group. (d, e) Immunohistochemical analysis of collagen I with aging. (f, g) Immunohistochemical analysis of *α*-actin with aging. The data are presented as the means ± SD for *n* = 3 per group. ^∗^*P* < 0.05 and ^∗∗^*P* < 0.01 compared with the 16-month-old group.

The smooth muscle layer of the CC thinned with aging, and the thinning was accompanied by the development of discontinuous and disordered cavernous sinuses; these changes became more severe with aging (Figure 3(a)). The relative quantities of SMCs and collagen were determined by Masson staining. The SMC/collagen ratios were 0.30 ± 0.06 in the 4-month-old group, 0.28 ± 0.02 in the 8-month-old group, 0.21 ± 0.07 in the 12-month-old group, 0.19 ± 0.02 in the 16-month-old group, 0.14 ± 0.04 in the 20-month-old group, and 0.08 ± 0.005 in the 24-month-old group. Among the groups, the 16-month-old group had a significantly lower SMC/collagen ratio than the 8-month-old group, while the ratio of the 24-month-old group was the lowest (Figures 3(b) and 3(c), *P* < 0.05). Similarly, immunohistochemistry showed that the protein levels of *α*-actin declined with aging (Figures 3(d) and 3(e), *P* < 0.05), while those of collagen I increased (Figures 3(f) and 3(g), *P* < 0.05), especially in the 8-month-, 16-month-, and 24-month-old groups.

### 3.4. Identification and Analysis of DEGs in ED Samples via Bioinformatics Analysis

**Figure 4:**
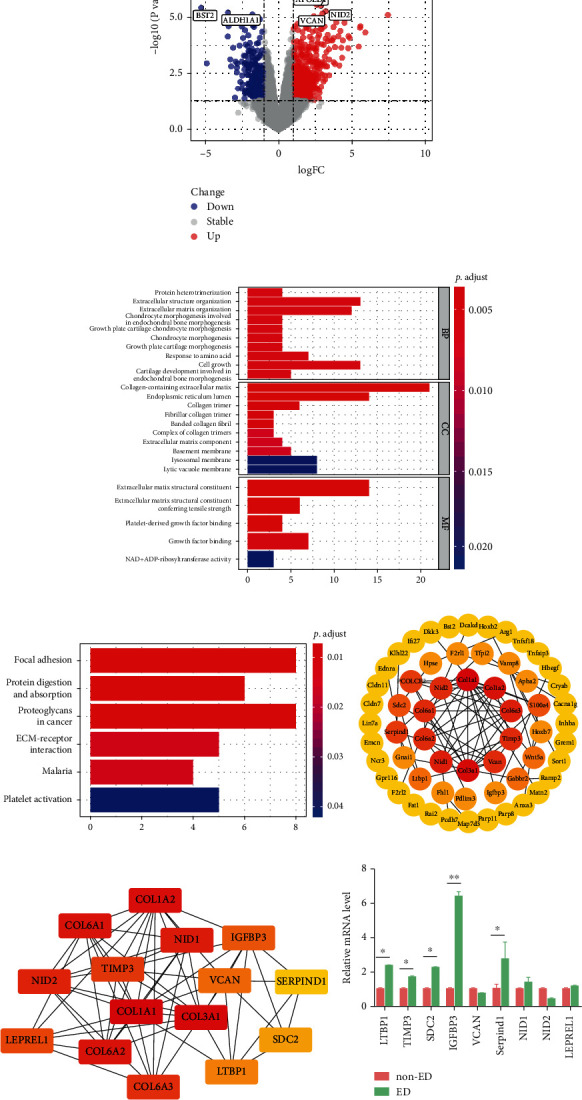
Bioinformatics analysis of ED/non-ED samples. (a) Volcano plot analysis of DEGs between ED and non-ED samples. (b, c) KEGG pathway and GO enrichment analyses of the DEGs. (d) The PPI networks were constructed using Cytoscape. (e) The hub genes were ranked with Cytoscape using the MCC method. (f) The mRNA expression levels of the hub genes were measured by qRT–PCR between in the CC of rats with and without ED. ^∗^*P* < 0.05 and ^∗∗^*P* < 0.01 compared with the non-ED group.

A total of 116 DEGs were identified, including 35 downregulated genes and 81 upregulated genes (Figure 4(a)). The enriched functions and pathway terms of the DEGs included the extracellular matrix (ECM) and structure organization, collagen-containing ECM, ECM constituent, focal adhesion, protein digestion and absorption, proteoglycans in cancer, ECM-receptor interaction, malaria, and platelet activation terms (Figures 4(b) and 4(c)). The constructed PPI networks of the DEGs are shown in Figure 4(d). We selected fifteen hub genes, including COL1A1, COL3A1, COL1A2, COL6A1, COL6A2, NID1, NID2, COL6A3, TIMP3, LEPREL1, IGFBP3, VCAN, LTBP1, SDC2, and SERPIND1, with the cytoHubba plugin of Cytoscape. The results revealed that these genes were principally associated with cellular responses to ECM structural constituents (Figure 4(e)). The hub genes were verified to be differentially expressed in the CC tissues of rats with age-related ED compared with those of non-ED rats; among them, IGFBP3 was remarkably upregulated (Figure 4(f)).

### 3.5. The Expression of IGFBP3 in the CC Was Upregulated with Aging

**Figure 5:**
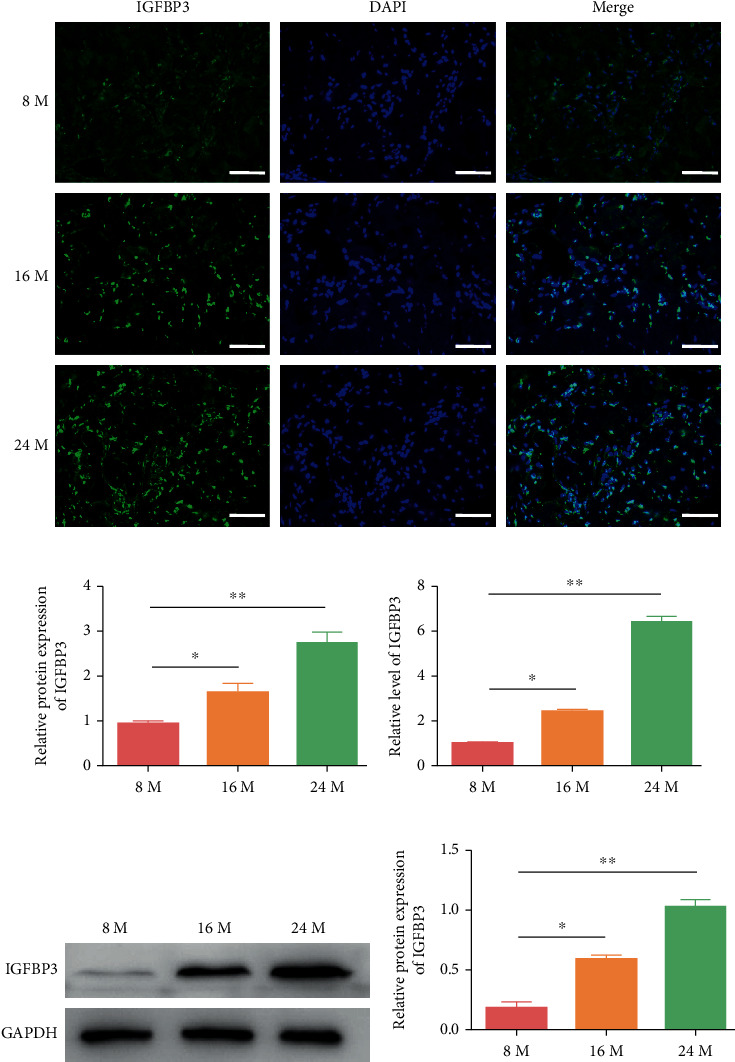
Verification of hub gene expression. (a, b) Immunofluorescence of IGFBP3 in the CC in age-related ED. Green represents IGFBP3. Cell nuclei were stained with DAPI (blue). (c) The mRNA expression levels of IGFBP3 were measured by qRT–PCR. (d, e) The protein expression levels of IGFBP3 were detected by western blot analysis. The data are represented as the means ± SD for *n* = 3 per group. ^∗^*P* < 0.05 and ^∗∗^*P* < 0.01 compared with the 16-month-old group.

To determine the expression of IGFBP3 in CC with aging, the expression levels of IGFBP3 were detected by immunofluorescence in the 8-month-, 16-month-, and 24-month-old groups (Figure 5(a)). The results indicated that IGFBP3 was significantly upregulated with aging (Figure 5(b), *P* < 0.05). Afterward, the expression of IGFBP3 in CC in the 8-month-, 16-month-, and 24-month-old groups was detected by qRT–PCR (Figure 5(c), *P* < 0.05) and western blot analysis (Figures 5(d) and 5(e), *P* < 0.05). The results were consistent with the immunofluorescence results, strongly suggesting that IGFBP3 was significantly upregulated with aging.

### 3.6. Downregulation of IGFBP3 in CCSMCs Enhanced Cell Viability and Proliferation

**Figure 6:**
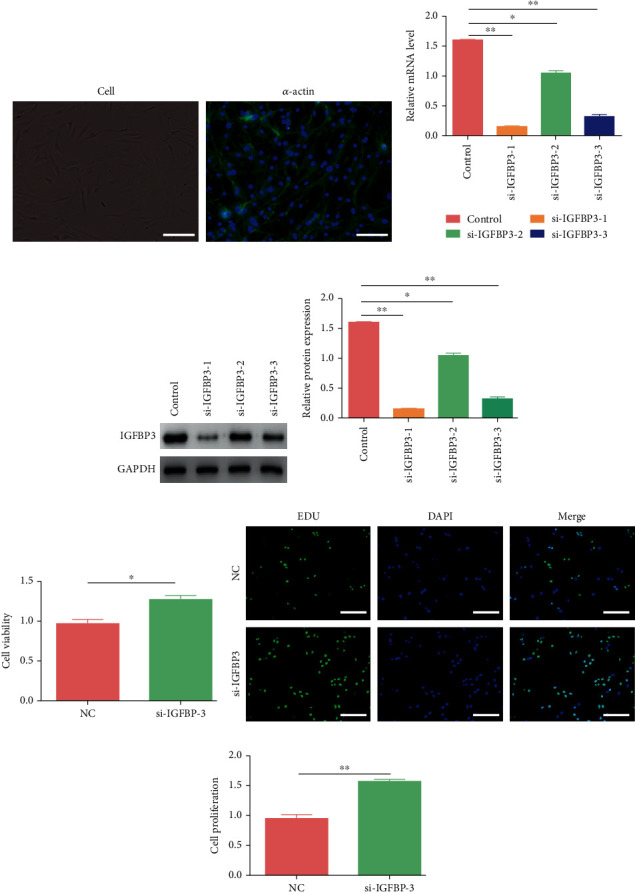
Knockdown of IGFBP3 expression in CCSMCs enhanced cell viability and proliferation. (a) Morphology and immunofluorescence of *α*-actin in cells (magnification 400x). Green represents *α*-actin. (b) IGFBP3 mRNA expression was knocked down with siRNA in CCSMCs. (c, d) The protein expression levels of IGFBP3 in cells with siRNA-mediated knockdown were detected by western blot analysis. (e–g) The viability and proliferation of CCSMCs among the groups were assessed by using CCK-8 assays and EdU staining. The data are represented as the means ± SD for *n* = 3 per group. ^∗^*P* < 0.05 and ^∗∗^*P* < 0.01 compared with the control group.

CCSMCs were identified via morphology and molecular markers. Inverted phase-contrast microscopy revealed spindle-shaped cells, which were further verified according to *α*-actin expression via immunofluorescence (Figure 6(a)). To conduct loss-of-function experiments, we successfully downregulated IGFBP3 expression in CCSMCs with siRNAs targeting IGFBP3 (Figures 6(b)–6(d), *P* < 0.05). The cell viability and proliferation of IGFBP3-deficient CCSMCs were markedly greater than those of control cells, as determined by EdU staining and the CCK-8 assay (Figures 6(e)–6(g), *P* < 0.05).

### 3.7. The Apoptosis Levels of CCSMCs Were Reduced after IGFBP3 Knockdown

**Figure 7:**
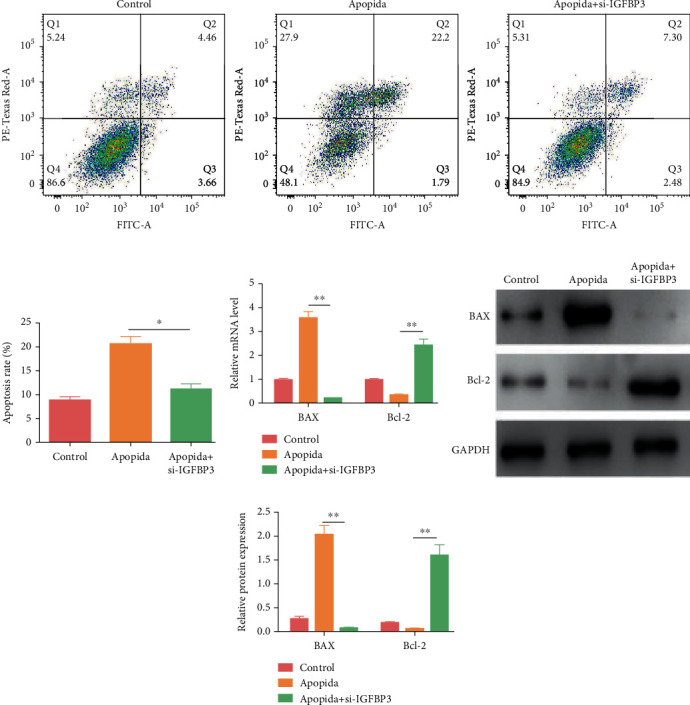
Knockdown of IGFBP3 attenuated CCSMC apoptosis. (a) Apoptosis was determined by flow cytometry. (b) Quantitative analysis of the apoptotic rate. The data are presented as the means ± SD (*n* = 3). (c) The mRNA expression levels of Bcl-2 and Bax were measured by qRT–PCR. (d, e) The protein expression levels of Bcl-2 and Bax were detected by western blot analysis. Apopida is an apoptosis inducer. ^∗^*P* < 0.05 and ^∗∗^*P* < 0.01 compared with the apopida group.

FITC-Annexin V/PI double staining was used with flow cytometry to analyze the potential antiapoptotic effects of IGFBP3 downregulation in CCSMCs (Figure 7(a)). The results demonstrated that the apoptotic rate was significantly decreased following treatment with siRNA (Figure 7(b), *P* < 0.05). The expression levels of Bcl2 and Bax after 48 h of coculture with siRNA were determined by qRT–PCR and western blot analysis (Figures 7(c)–7(e), *P* < 0.05). The results showed that the mRNA and protein levels of Bax were markedly lower in the IGFBP3 deficiency group than those in the control group, while those of Bcl-2 were significantly higher.

### 3.8. The Expression of Fibrosis-Related Markers in CCSMCs Was Attenuated after IGFBP3 Knockdown

**Figure 8:**
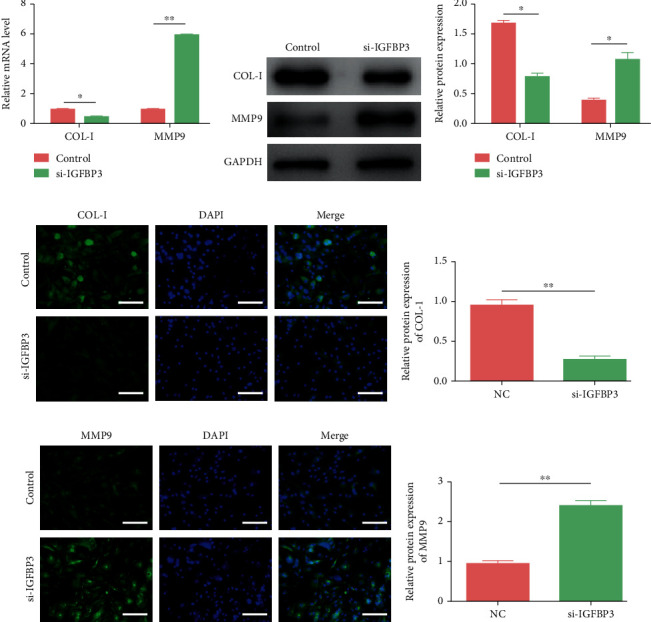
Knockdown of IGFBP3 inhibited CCSMC-related fibrosis. (a) qRT–PCR analysis of collagen I and MMP9 in CCSMCs from each group. (b, c) Western blot analysis of collagen I and MMP9 in CCSMCs from each group. (d, e) Immunofluorescence analysis of collagen I in each group. (f, g) Immunofluorescence analysis of MMP9 in each group. ^∗^*P* < 0.05 and ^∗∗^*P* < 0.01 compared with the control group.

After IGFBP3 knockdown, CCSMCs were subjected to qRT–PCR, western blotting, and immunofluorescence to investigate the expression of fibrosis-related markers. As shown in Figure 8(a), the mRNA levels of collagen I were markedly lower in the IGFBP3-deficient CCSMC group than those in the control group, while those of MMP9 were significantly higher (*P* < 0.05). Western blot analysis showed that the protein levels of collagen I and MMP9 were consistent with the above findings (Figures 8(b) and 8(c), *P* < 0.05). Immunofluorescence analysis showed that collagen I expression was obviously lower in the siRNA-treated group than that in the control group (Figures 8(d) and 8(e), *P* < 0.05), while MMP9 expression was significantly higher (Figures 8(f) and 8(g), *P* < 0.05).

### 3.9. The OS Levels of CCSMCs Were Diminished after IGFBP3 Knockdown

**Figure 9:**
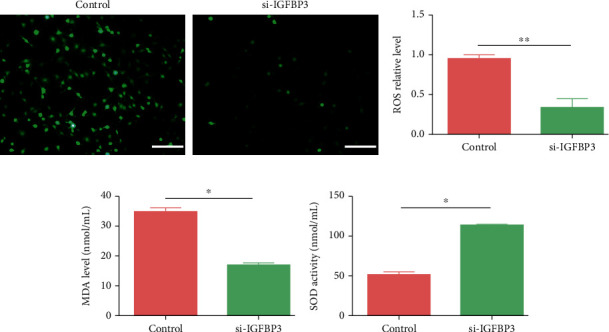
Knockdown of IGFBP3 attenuated CCSMC-related OS. (a, b) ROS production was detected by fluorescence analysis. (c, d) MDA levels and SOD activity were detected with relevant test kits. ^∗^*P* < 0.05 and ^∗∗^*P* < 0.01 compared with the control group.

To further investigate the relationship between IGFBP3 and OS in CCSMCs, we investigated the effects of IGFBP3 silencing with siRNA on the generation of ROS and the activity of MDA and SOD. As shown in Figures 9(a) and 9(b), ROS production was lower in the IGFBP3-knockdown CCSMCs than in the control CCSMCs, while SOD activity was significantly higher (Figure 9(c), *P* < 0.05) and MDA levels were markedly lower (Figure 9(d), *P* < 0.05).

## 4. Discussion

**Figure 10:**
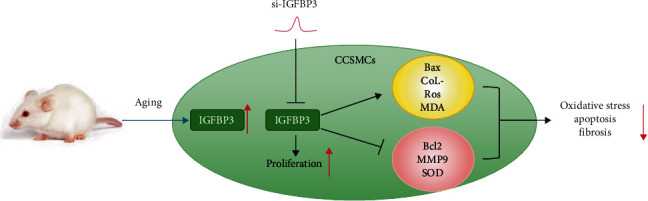
Potential mechanisms by which IGFBP3 participates in age-related ED. The expression of IGFBP3 increases with aging. Downregulation of IGFBP3 with siRNA in CCSMCs enhances cell proliferation while reducing apoptosis, fibrosis, and OS, as determined by downregulation of Bax, collagen I, ROS, and MDA and upregulation of Bcl-2, MMP9, and SOD.

This study is aimed at investigating the potential key molecular mechanism involved in age-related ED. The erectile response decreased with aging, while the SMC/collagen ratio of the CC declined, suggesting that the SMC content decreased and fibrosis increased during the aging process. These changes were accompanied by increased apoptosis and OS levels. To further clarify the corresponding gene level changes in ED, we selected appropriate data for bioinformatics analysis. Functional analysis showed that these DEGs were markedly enriched in ECM-associated changes and focal adhesion, which triggers phosphatidylinositol 3-kinase (PI3K) and mitogen-activated protein kinase (MAPK) signaling pathways that affect proliferation and apoptosis [16]. Fifteen hub genes were then confirmed; among them, IGFBP3 exhibited significantly enhanced expression in the CC with aging. Furthermore, aged CCSMCs were isolated and transfected with siRNA to knock down the expression of IGFBP3. Subsequent experiments showed that the viability and proliferation of CCSMCs were enhanced. Subsequently, collagen I was downregulated, while MMP9 was upregulated. Additionally, the levels of Bax, MDA, and ROS in CCSMCs were downregulated; however, the levels of Bcl2 and SOD were upregulated, indicating the attenuation of apoptosis and OS (shown in Figure 10). These results demonstrate that IGFBP3 may be a bridge between aging and ED.

Insulin-like growth factors (IGFs) and IGF binding proteins (IGFBPs) are the main components of the IGF regulatory system, which is tissue-specific in each organ; however, all versions of this system share similar components, including specific ligands, IGFBPs (no. 1–8), IGF receptors (types I and II), and IGFBP-specific proteases. IGFs in biological fluids are associated with IGFBPs, which are the principal regulators of IGF bioactivity and activity in metabolic signaling pathways. Moreover, these proteins extend the half-lives of IGFs in the bloodstream, store IGFs in specific tissue compartments, inhibit the activity of IGFs by lowering the accessibility of their receptors, and protect IGFs from proteolytic degradation. Among IGFBPs, IGFBP3 is the major carrier of IGF-1 and regulates its biological activity with the highest content and strongest binding ability [17]. In addition to exerting functions via activation in an IGF-dependent manner, IGFBP3 can also play roles in IGF-independent actions [18].

IGFBP3 plays crucial roles in multiple etiologies of ED. Zhou et al. found that IGFBP3 is upregulated in rats with diabetic ED and that IGFBP3 downregulation can improve erectile function by increasing IGF-1 bioavailability and cavernous cyclic guanosine monophosphate (cGMP) concentrations [19]. They also demonstrated that IGFBP3 short hairpin RNA (shRNA) might restore erectile function by decreasing the concentrations of serum low-density lipoprotein and triglyceride, increasing the percentage of CCSMCs, and improving nitric oxide-cGMP signaling activity in rats with streptozotocin-induced diabetes [20]. In one study, IGFBP3 was found to be upregulated in two-kidney one-clip hypertensive rats treated with the *β*-blocking agent propranolol compared with control rats, and this upregulation was accompanied by downregulation of cGMP and erectile function [21]. Our study showed that IGFBP3 levels increased with aging and that IGFBP3 modulated CCSMC-related proliferation, apoptosis, fibrosis, and OS. Thus, it can be speculated that IGFBP3 is involved in multiple etiologies of ED, including age-related ED, and may be one of the common causes of ED.

IGFBP3 was upregulated in the CC with aging, suggesting that it may be a senescence-associated gene involved in age-related ED. IGF signaling pathways are among the most conserved aging pathways in organisms ranging from yeast to mammals. During aging, notable increases in the mRNA and protein expression of IGFBP3 have been observed in human umbilical vein endothelial cells (HUVECs), while knockdown of IGFBP3 in old cells with an shRNA-expressing retrovirus results in partial attenuation of a variety of senescence phenotypes. In contrast, upregulation of IGFBP3 in young cells accelerates cellular senescence, as confirmed by cell proliferation analysis and SA-beta-gal staining [22]. Debacq-Chainiaux et al. analyzed the triggering of premature senescence by sublethal concentrations of tert-butyl hydroperoxide and ethanol in WI-38 human diploid fibroblasts by DNA array and revealed that IGFBP3 is one of 16 senescence-related genes that regulate the appearance of several biomarkers of senescence [23]. Xiao et al. successfully induced HEK293 cell senility via G protein-coupled receptors (GPCRs) and found through qRT–PCR that IGFBP3 was upregulated significantly [24]. The upregulated IGFBP3 in senescent cells can also be secreted into conditioned medium via a senescence-associated secretory phenotype (SASP) and thus appears to be a senescence-mediating factor in young cells, indicating that IGFBP3 may be the trigger of senescence [25]. In HTRA4-treated cells, colorectal expression of senescence genes, including IGFBP3, SERPINE1, and SERPINB2, is significantly upregulated, which promotes senescence [26]. Hence, we suggest that IGFBP3 is a key gene involved in aging processes, including age-related ED.

IGFBP3 participates in the regulation of CCSMC apoptosis to modulate the CCSMC content via autocrine and paracrine mechanisms. Mofid et al. found that IGFBP3 resensitizes chemoresistant pancreatic ductal adenocarcinoma cells by activating apoptosis through recruitment of its death receptor (IGFBP3R), as indicated by Bcl-2 downregulation, Bax upregulation, and caspase-3 and caspase-8 activation [27]. Wang et al. found that sanguinarine promotes hepatocellular carcinoma by inhibiting the expression of Bcl-2 and promoting the expression of Bax and caspase-3 via upregulation of IGFBP3 expression [28]. IGFBP3 can enhance mitochondrial-dependent apoptosis of tumor tissues by increasing ROS production through NF-*κ*B activation and cytokine production [29]. Endogenous IGFBP3 can also play a role in intrinsic apoptosis by facilitating phosphorylation and nuclear export of Nur77 to the cytoplasm, where it exerts its apoptotic effect [30]. Accumulation of IGFBP3 sequesters IGF1 away from the type I IGF receptor (IGF-1R), leading to apoptosis [31, 32], which consequentially reduces cell survival by blocking the PI3K and MAPK signaling pathways [33]. IGFBP3 is a potent antiproliferative and proapoptotic factor in cancer cells whose biological action is mediated through interactions with a variety of binding partners on the cell surface and within cells via multiple signaling pathways. Our study shows that upregulation of IGFBP3 enhances the apoptosis of aged CCSMCs and that downregulation of IGFBP3 rescues CCSMCs from apoptosis, which probably impacts the apoptosis levels of the CC.

In the current study, IGFBP3 was closely related to CC fibrosis, similar to the findings of a study on the relationship between IGFBP3 and tumor vascular fibrosis conducted by Hess et al. [34]. Arab et al. demonstrated that IGFBP3 expression is increased in patients with alcoholic hepatitis and that related hepatic stellate cell activation promotes alcohol-induced steatohepatitis [35]. In addition, the IGF-1/intact IGFBP3 ratio is reduced in patients with liver fibrosis [36]. Another study has shown that miR-34/449 might contribute to airway inflammation and fibrosis by modulating IGFBP3-mediated autophagy activation [37]. IGFBP3 plays a role in fibrosis and ECM deposition in idiopathic pulmonary fibrosis [38]. Upregulation of endogenous IGFBP3 and TGF-*β*1 expression enhances excess collagen I*α*I production in SMCs and contributes to fibrosis and stricture formation in Crohn's disease via a TGF-*β*RII/I-dependent and Smad2/3-dependent mechanism [39]. MMPs form a key family of proteolytic enzymes that participate in tissue remodeling by degrading ECM and basement membrane components. Chen et al. revealed that MMP9 expression in aortic SMCs can be induced by Ang II and that overexpression of IGFBP3 can reduce the expression of MMP9 [40]. Suppressing IGFBP3 expression in mice with diabetic cardiomyopathy can alleviate cardiac fibrosis and enhance cell proliferation while inducing AKT and ERK phosphorylation [41]; this potentially plausible signaling pathway needs to be explored further. In this study, we found that downregulation of IGFBP3 in CCSMCs contributed to remarkable downregulation of collagen I and upregulation of MMP9 in the knockdown group compared with the control group, suggesting that IGFBP3 may be able to be targeted to regulate CCSMC fibrosis.

Increased OS has been acknowledged as a crucial factor for the progression of cellular senescence and aging [42]. In proximal tubular epithelial cells (PTECs) of the kidneys, IGFBP3 can mediate HG-induced apoptosis by elevating OS [43]. Huang et al. demonstrated a connection of IGFBP3 to OS and hypoxia; namely, that IGFBP3 gene upregulation is hypoxia-inducible factor (HIF) 1*α*-dependent and ROS-dependent [31]. ROS are natural byproducts of oxygen metabolism in cells. MDA is a toxic molecule produced by ROS due to lipid peroxidation of the cellular membrane that represents the degrees of lipid peroxidation and OS. SOD can remove superoxide radicals, but its activity can be inhibited by excess ROS. We found that OS levels increased with aging in the CC, but the interference with IGFBP3 rescued CCSMCs from OS, as verified by antioxidant (SOD) and oxidant (ROS and MDA) assays. These findings indicate that IGFBP3 is involved in modulating OS in CCSMCs.

## 5. Conclusion

Collectively, the data indicate that the penile erectile response of SD rats declines with aging. This decline is accompanied by enhanced CC fibrosis, apoptosis, and OS and a decreased smooth muscle/collagen ratio. Upregulation of IGFBP3 plays an important role in this process; furthermore, downregulation of IGFBP3 improves the viability and proliferation of CCSMCs and alleviates cell fibrosis, apoptosis, and OS. Thus, IGFBP3 is a potential therapeutic target for age-related ED.

## Figures and Tables

**Table 1:** Primer sequences used for qRT–PCR.

Genes	Primer sequences (5′→3′)
NID1	F:ACCAGGGTGTGTGGGTGTTT
	R:CCAGGCCGAGATGAGAGGTT
NID2	F:ACGCTTCTACGATGCCCAGT
	R:GCGATGGCTGGGAAGTCAGT
TIMP3	F:CGGGCCAAAGTGGTGGGAAA
	R:CTAGCTTAAGGCCACAGAGACT
LEPREL1	F:TACCTGCAGAGGGCCTACAT
	R:TGTCTACTAACGGGGCTTCC
IGFBP3	F:GGAGGACCACAATGCTGGGA
	R:TCTGGGTGTCTGTGCTCTGG
VCAN1	F:ACCCACCTGTTAAAGGCTCT
	R:GCCACCAGGACAGTAGTCTC
LTBP1	F:CAGCAGCAAAGCGTACTTCAAC
	R:GGGTGCCACTTGAGAATGTATC
SDC2	F:TAGTGCTGCTCCCGAAGTGG
	R:GGGTCCTGCTTTTCTTCTGCC
SERPIND1	F:ACGACTACCTGGACCTGGAG
	R:GGACTCGGTAAAGGTTGAAGGC
*α*-actin	F:GGCCGAGATCTCACCGACTA
	R:GCAGCAGTGGCCATCTCATT
Collagen I	F:TCCCAAAGGCAACAGTGGT
	R:CCACGGGCTCCTCGTTTTCC
MMP9	F:CACGACAGCTGACTACGACAC
	R:GCAGGCAGAGTAGGAGTGG
MMP2	F:TCGCCCATCATCAAGTTCCC
	R:TGCATCTTCTTGAGGGTGTCC
Bcl-2	F:CAGCCTGAGAGCAACCGAAC
	R:CGACGAGAGAAGTCATCCCC
Bax	F:TTTCATCCAGGATCGAGCAG
	R:TGTTGTCCAGTTCATCGCCAA
GADPH	F:CCTTCCGTGTTCCTACCCCC
	R:TAGCCCAGGATGCCCTTTAG

## Data Availability

The data of the materials and methods and results to support the conclusions are included in this article. If any other data are needed, please contact the corresponding author.

## Abbreviations

COL1A1: Collagen type I alpha 1 chain
COL3A1: Collagen type III alpha 1 chain
COL1A2: Collagen type I alpha 2 chain
COL6A1: Collagen type VI alpha 1 chain
COL6A2: Collagen type VI alpha 2 chain
NID1: Nidogen 1
NID2: Nidogen 2
COL6A3: Collagen type VI alpha 3 chain
TIMP3: Tissue inhibitor of metalloproteinases-3
LEPREL1: Leprecan-like 1
IGFBP3: Insulin-like growth factor binding protein 3
VCAN: Versican
LTBP1: Latent transforming growth factor beta binding protein 1
SDC2: Syndecan 2
SERPIND1: Serpin family D member 1
GAPDH: Glyceraldehyde-3-phosphate dehydrogenase
SDS-PAGE: Sodium dodecyl sulfate-polyacrylamide gel electrophoresis
PVDF: Polyvinylidene fluoride.
